# Autophagy, an accomplice or antagonist of drug resistance in HCC?

**DOI:** 10.1038/s41419-021-03553-7

**Published:** 2021-03-12

**Authors:** Yafei Wu, Jigang Zhang, Qin Li

**Affiliations:** Department of Clinical Pharmacy, Shanghai General Hospital, Shanghai Jiao Tong University School of Medicine, Shanghai, China

**Keywords:** Cancer metabolism, Cancer therapeutic resistance

## Abstract

Hepatocellular carcinoma (HCC) is a highly lethal malignancy characterized by poor prognosis and a low 5-year survival rate. Drug treatment is proving to be effective in anti-HCC. However, only a small number of HCC patients exhibit sensitive responses, and drug resistance occurs frequently in advanced patients. Autophagy, an evolutionary process responsible for the degradation of cellular substances, is closely associated with the acquisition and maintenance of drug resistance for HCC. This review focuses on autophagic proteins and explores the intricate relationship between autophagy and cancer stem cells, tumor-derived exosomes, and noncoding RNA. Clinical trials involved in autophagy inhibition combined with anticancer drugs are also concerned.

## Facts

- Although there is a basal level of autophagy in cells, cellular stressors including chemotherapy can induce tumor cell autophagy.
- Antitumor drugs cause changes in the expression or activity of autophagy-related proteins, thus affecting the autophagy of HCC.
- Autophagy activation promotes tumor cell survival with anticancer drugs.

## Open questions

- How to reverse tumor resistance by autophagy?
- How to explore the mechanism of the difference of autophagy induced by different anti-tumor drugs?
- How to use autophagy-related proteins to design drug targets and improve the efficacy of antitumor drugs?
- How to optimize the combination of autophagy inhibition and antitumor drugs?

## Introduction

HCC accounts for the major subtype of liver cancer, which is the second roughly dead tumor ranking after pancreatic cancer^1^. HCC treatments include surgical resection, radiofrequency ablation, liver transplantation, transarterial chemoembolization, chemotherapy, targeted therapy, and immunotherapy, while the 5-year survival rate of HCC is eighteen percent^2,3^. Even so, only a few patients are sensitive to anticancer drugs, and most patients with intermediate- or advanced-stage HCC fail to respond to anticancer drugs efficiently. Drug resistance has already occurred and a reduction in the overall survival rate of HCC happened in HCC patients^4^. Therefore, elucidating the drug-resistance mechanism of HCC, finding drug-resistance targets, and optimizing the treatment plan is of great significance to rescue HCC patients.

The mechanism of drug resistance is very extensive, extending from the level of tumor cells to cancer stem cells (CSCs). Autophagy, the complex mechanism of transferring cellular substances to lysosomes for degradation, involves drug resistance and causing treatment failure in HCC^5^. Long noncoding RNA (lncRNA) and microRNA promote the further development of the mechanism of drug resistance^6^. CSCs help resist the toxic effects of anticancer drugs and promote the development of drug resistance with self-renewal and unlimited proliferation ability in HCC^7^. Interestingly, findings have confirmed the profound significance of CSCs, noncoding RNA (ncRNA), and tumor-derived exosomes in the regulation of autophagy affecting drug resistance^6,8,9^. This review focuses on the relationship between autophagy and HCC drug resistance, especially in sorafenib, doxorubicin, platinum drugs, and immune checkpoint inhibitors. The interaction between autophagy and CSCs, tumor-derived exosomes, and ncRNA in drug resistance of HCC is also concerned. What is more, clinical trials involved in autophagy inhibition combined with anticancer drugs are also mentioned.

## Autophagy and autophagy-related proteins in HCC

Autophagy includes autophagosome formation, autophagosome maturation, autolysosome formation, and cargo degradation in order of precedence involved in maintaining the balance of cell component synthesis and decomposition^10^. When the cell is in a nutritionally deficient state, autophagy degrades the macromolecules through the lysosomal pathway and participates in maintaining the nitrogen balance and the homeostasis of the cell environment^11^. Autophagy can be divided into microautophagy, macroautophagy, and chaperone-mediated autophagy according to the transport pathways^12–16^ and can be divided into mitophagy, ribophagy, pexophagy, reticulophagy, and xenophagy via the ability to selectively degrade cargo^17–19^. The substrate selection mechanism of selective autophagy is unclear, and ubiquitination may play an important role in it^20^.

**Fig. 1:**
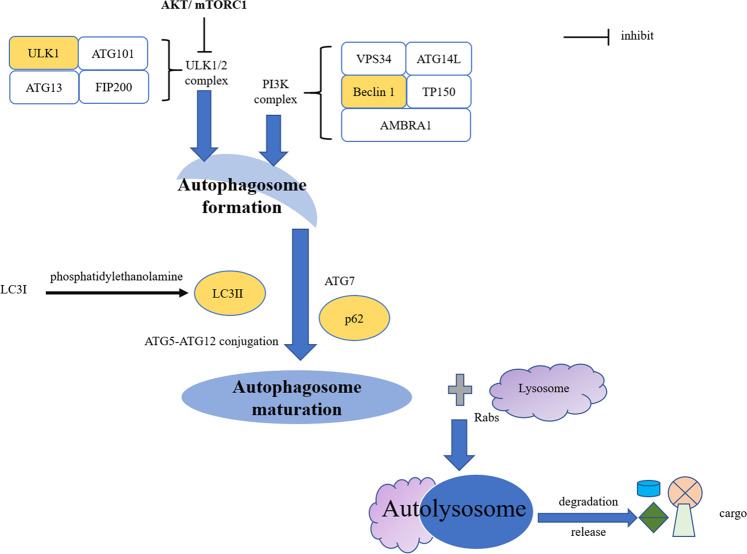
Autophagy and autophagic proteins. Autophagy is roughly summarized as autophagosome formation, autophagosome maturation, and autolysosome formation. At the initial stage of autophagy formation, ULK1/2 complex is composed of ULK1, ATG101, ATG13, and FIP200. Subsequently, the PI3K complex with Beclin 1 as the core is added to promote the formation of the autophagosome. Then, LC3-I was transformed into LC3-II with phosphatidylethanolamine, ATG5–ATG12 conjugation, and p62 recruitment to promote the maturation of autophagosomes. Finally, the autophagosome fuses with lysosomes under the mediation of Rabs and performs the degradation and release of cargo.

Regardless of the way in which autophagy is classified, autophagy-related proteins, such as Unc-51-like autophagy-activating kinase (ULK), Beclin 1, microtubule-associated protein 1 light chain 3 (LC3), and p62 largely overlap (Fig. 1). ULK1/2 complex includes ULK1, autophagy-related gene 13 (ATG13), ATG101, and FIP200, and phosphatidylinositol 3-kinase (PI3K) complex, including VPS34, TP150, Beclin 1, ATG14L, and AMBRA1, participates in autophagosome formation. ATG7, ATG5–ATG12 conjugation, LC3, and p62 were involved in the maturation of the autophagosome. Lysosomes, under the joint action of Rabs, SNAREs, and tethers, fuse with autophagosomes and participate in the degradation of cargo, then release nutrients back into the cytoplasm^21^.

### ULK in HCC autophagy

ULK, a serine/threonine kinase homologous to yeast ATG1, is the core regulator molecule of the autophagy initiation step. ULK complex promotes the formation of autophagosomes under an activated condition. Activation of the ULK complex is also a complex process, including phosphorylation, ubiquitination, and acetylation of various autophagy-related proteins. Among them, mammalian target of rapamycin complex 1 (mTORC1) and AMP-dependent protein kinase (AMPK) directly participate in regulating ULK activation^22^; AMBRA1–TRAF6 complex, p32, and USP24 participate in ULK ubiquitination, which are all related to the stability of ULK; TIP60 participates in UKL acetylation, and OGT participating in UKL glycosylation process is related to ULK activity. Activating ULK not only affects the initial process of autophagosome formation, but also has great significance in activating p62-mediated selective autophagy and regulating caspase3 to affect cell apoptosis. In recent years, the role of ULK in HCC is also widely advertised: UKL1 combined with LC3B can improve the accuracy of prognostic assessment of patients^23^. Besides, modifying the Lys46, Tyr94, and Asp165 amino acid residues of ULK1 negatively regulates autophagy and effectively induces apoptosis. The development of inhibitors against ULK affecting tumor autophagy is a very promising direction, and licorice chalcone A or Glycyrrhizin A is a paradigm^24^. Furthermore, XST14 as an inhibitor of ULK1 combined with sorafenib can produce excellent synergistic effects on HCC both in vivo and in vitro^25^.

### Beclin 1 in HCC autophagy

Beclin 1, a highly homologous gene of yeast ATG6, participates in the formation of autophagosome by binding with VPS15, VPS34, and ATG14^21^. Phosphorylation or ubiquitination on Beclin 1 residues can affect the role of autophagy in tumor survival and apoptosis^26–28^. Although K32 and K263 ubiquitination improves Beclin 1 stability and self-dimerization, thereby inhibiting autophagy, Beclin 1 phosphorylation by ULK1 seems to promote autophagy^29,30^. NO reduces Beclin 1 binding to VPS34 and increases the interaction between BCL-2 and Beclin 1, thus inhibiting autophagy and promoting apoptosis^31^. Notedly, under normal circumstances, the combination of BCL-2 and Beclin 1 can inhibit the activity of Beclin 1, and autophagy is maintained at a normal physiological level. However, nutrient starvation triggers BH3-only proteins (a member of the BCL-2 family) activation or BCL-2 phosphorylation, which induces autophagy through the dissociation of BCL-2 and Beclin 1 complex and leads to poor prognosis^32^. Furthermore, BCL-2 proteins, which are known for their dual role in apoptosis and autophagy, have also been demonstrated to be a poor predictor for prognosis and play a key role in chemoresistance in HCC^32–35^. Other studies have demonstrated that p53 inhibitor and AKT activator depressing the expression of Beclin 1 caused autophagy inhibition^36,37^.

In addition, in-depth studies on Beclin 1 have revealed its role in the prognosis, proliferation, metastasis, and drug resistance of HCC. The expression level of Beclin 1 in HCC tissues is lower than that in adjacent tissues by a tissue microarray research^38^. These results are not inconsistent with the conclusion that Beclin 1 can be used as a prognostic indicator^39^. Similarly, a meta-analysis of 1124 HCC patients identified Beclin 1 as a prognostic marker based on the negative correlation between Beclin 1-positive expression and alpha-fetoprotein, cirrhosis, and vascular invasion^40^. When the interaction between BCL-2 and Beclin 1 was blocked, autophagy was induced, further inhibiting the proliferation and migration of HepG2 cells^41^. The situation is more complicated when drug resistance is involved. Beclin 1 mediates autophagy in HCC sorafenib and regorafenib resistance, but the specific mechanism is still being explored.

### LC3 in HCC autophagy

LC3, a homolog of yeast ATG8, including LC3-I and LC3-II, is mainly located on the surface of preautophagic vesicles and autophagic vesicles^42^. LC3-I is mainly expressed under physiological conditions, and when autophagy is activated, LC3-I is conjugated to phosphatidylethanolamine to form LC3-II and localized on the autophagosome membrane^43^. In the late stage of autophagosome formation, LC3 involves in the expansion and shutdown of the autophagy membrane, and the conjugation of ATG12 and ATG5 cannot be ignored^21^. Immunohistochemical testing of 535 HCC samples and adjacent nontumor (ANT) tissues revealed that the high LC3 expression in the tumor and liver microenvironments is significantly associated with lower HCC recurrence^44^. A meta-analysis involving 949 patients in HCC indicated that positive LC3 expression was related to the size of the tumor and the occurrence of HCC^45^. However, the combination of high Axl and low LC3 expression could significantly predict poorer prognosis for HCC patients who underwent hepatectomy in HCC^46^. Other data demonstrate that the complex involvement of TIPRL/LC3/CD133 in HCC aggressiveness can serve as potential biomarkers for early detection in a combined model or worked individually^47^. The above studies have demonstrated the potential of LC3 as a biomarker for early detection and early warning, but many challenges remain. A great deal of data and in-depth research are needed to support LC3 or combine it with other biomarkers to achieve a more accurate detection in patients with different disease processes. Of course, there is a need for further optimization of the assay for improving patient compliance and clinical value.

### P62 in HCC autophagy

Ubiquitin-binding protein p62, also known as sequestosome1 (SQSTM1), is a scaffold protein involved in a variety of cellular functions such as signal transduction, cell proliferation, cell survival, and tumorigenesis. When autophagy is defective, p62 is accumulated, while active autophagy leads to p62 degradation^48^. In addition, p62 is also a receptor protein for ubiquitin degradation by selective autophagy, and during ubiquitin degradation, p62 binds autophagosomes and directs them to lysosomes^49^. Furthermore, continuous accumulation of p62 was proposed to participate in premalignant liver disease and most HCC. High expression of p62 led to the activation of Nrf2 and mTORC1 and initiating protection mechanism of HCC from oxidative stress-induced death^48,50^. HCC exhibits increased antioxidative response and survival rates in response to oxygen stress through phosphorylation of KHK-A-mediated p62’s aggregation^51^. Accumulation of phosphorylated p62 prevents Nrf2 degradation and results in its nuclear accumulation, which contributed to the growth of HCC and increased the anticancer activity of erastin and sorafenib in vitro and in HCC xenograft models^52,53^. Regulation of metabolic reprogramming by phosphorylated p62/Nrf2 promoted HCV- positive HCC proliferation and the tolerance of sorafenib and cisplatin^54^. However, p62 binding to different receptors involved in the occurrence of liver cancer as a negative regulator. P62, which downregulated in HCC-associated hepatic stellate cells (HSCs), interacted with vitamin D receptor and RXR and promoted their heterodimerization, related to HSC activity, fibrosis, and tumorigenesis^55^. Another study also demonstrated that the interaction between p62 and dead box protein 5 can inhibit liver tumorigenesis by stimulating autophagy^56^. Considering the different course of HCC, tumor heterogeneity, and the complexity of interactions between p62 and other proteins, the application of p62 as a potential target requires a large amount of data to support.

## Autophagy and drug resistance in HCC

Exhaustive data support that autophagy is associated with tumorigenesis, development, migration, and drug resistance^57,58^. However, there remains a question about how do autophagy flux and tumor cell status regulate autophagy to become a tumor killer or tumor guardian, especially in drug resistance^59,60^. Drug therapy promotes the occurrence of autophagy and induces a higher flux of autophagy, while increased autophagy flux further promotes drug resistance or causes tumor cell death with alterations of autophagy-related proteins^61,62^. In short, it is of profound significance to deepen the understanding of the mechanism of autophagy affecting drug resistance in HCC, including the first systemic drug, classical chemotherapeutic drugs, and novel antitumor drugs.

### Autophagy and sorafenib

As the first systemic drug approved by FDA, sorafenib plays an important role in the treatment of HCC as a multikinase inhibitor targeting serine/threonine kinases and receptor tyrosine kinases. Even if sorafenib has a good inhibitory effect on tumor angiogenesis and proliferation, many patients cannot escape the fate of drug resistance.

**Fig. 2:**
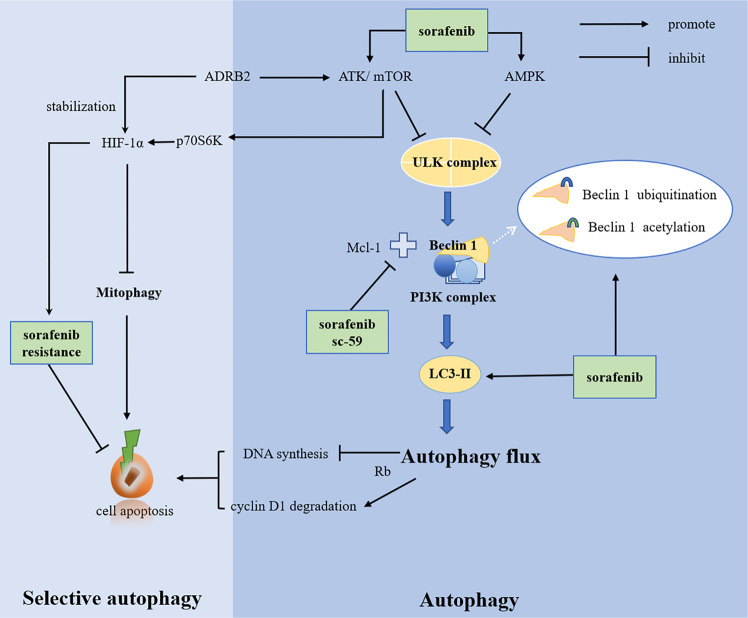
Sorafenib resistance and autophagy in HCC. The multikinase inhibitor sorafenib activates ATK/mTOR and AMPK signal to inhibit autophagy, causing autophagy-related resistance. Sorafenib influences the autophagy process by affecting the activity of various autophagy proteins. Sorafenib is involved in the chemical modification (ubiquitination and acetylation) of Beclin 1, which affects the activity of beclin1. In addition, sorafenib and its novel derivative, sc-59, inhibit the binding of Mcl-1 to Beclin 1. Autophagy links to apoptosis with the help of Rb protein, but sorafenib affects the formation of LC3-II, leading to autophagy inhibition and drug resistance that inhibit cell apoptosis.

Recently, the mechanisms of HCC sorafenib resistance remain ambiguous but may include overexpression of cytokines and activation of related signaling pathways, such as AKT, ERK, and AMPK; ncRNA and methylation-related epigenetic alterations; upregulation of ABC transporter and MDR expression and CSCs. The mechanisms also suggested that the tumor microenvironment, EMT, hypoxia, and autophagy are involved in the resistance to sorafenib, which, of course, is closely related to the secretion of cytokines and the activation of related signaling pathways^63–65^. However, the complex effects of sorafenib and autophagy on tumors are intriguing: sorafenib induces autophagy, promotes drug resistance, and keeps cell survival, and excessive autophagy leads to apoptosis. Important breakthroughs have been made in the study of sorafenib resistance and autophagy in HCC (Fig. 2).

#### Beclin1 involved in sorafenib resistance

Direct evidence suggests that sorafenib induces autophagy by upregulating the expression of Beclin 1 and modulating Beclin 1 acetylation levels makes HCC cells sensitive to sorafenib^66^. In addition to acetylation, Beclin 1 ubiquitination resulting in a stable inactive dimer-mediated autophagy inhibition is also associated with sorafenib sensitivity. There is solid evidence that large tumor suppressor kinase 1 (LATS1) restricts the autophagic apoptosis induced by sorafenib by promoting Beclin 1 ubiquitination at its lysine residues K32 and K263 (ref. ^29^). Besides chemical modification of Beclin 1, sorafenib also affects the binding of Beclin 1 to Mcl-1. Sorafenib and its new derivative sc-59 downregulated the phosphorylation level of signal transducer and activator of transcription 3 (STAT3), decreased the expression of Mcl-1, released the binding relationship between Beclin 1 and Mcl-1, and induced autophagy of PLC5 (a HCC cell line) in a time-- and dose-dependent manner^67^.

#### Signaling pathways in autophagy and sorafenib resistance

Sorafenib resistance is associated with activation of the AKT/mTOR and AMPK signaling pathway while linking to the recruitment and phosphorylation of ULK1 to repress autophagy^68–71^. Furthermore, ULK1 silencing or ULK1 inhibitor xst-14 combined with sorafenib significantly inhibited the malignant progression of HCC compared with sorafenib alone^25^. β-2 adrenergic receptor (ADRB2) signaling led to sorafenib resistance by negatively regulating autophagy with Beclin1/VPS34/ATG14 complex disruption, promoting the stability of hypoxia-inducible factor-1-α (HIF-1α) and the recombination of glucose metabolism^72^. Literature demonstrated that sorafenib induced endoplasmic reticulum (ER) stress and further induced autophagy with the redistribution of GFP-LC3-II and the accumulation of LC3-II on autophagosome membrane by virtue of the IRE1 signaling pathway, linking ER stress, autophagy, and apoptosis^73^. ER stress also regulated the expression level of Beclin 1 via PERK/ATF4 signaling pathway expanding the understanding of the relationship between autophagy and sorafenib^74^.

#### Mitophagy and sorafenib resistance

Mitophagy selects damaged mitochondria and controls mitochondrial homeostasis that is related to carcinogenesis and tumor progression^75^. In view of the relevant theories of hypoxia or reactive oxygen species (ROS) and hypoxia-induced drug resistance in tumors caused by the anti-angiogenesis of sorafenib, researchers focused on the relationship between sorafenib resistance and hypoxia/ROS-related mitophagy and made a certain breakthrough. The results have indicated that co-administration of melatonin and sorafenib induced an early mitophagic response in Hep3B cells and the status of mitochondrial membrane depolarization as a marker of ROS-related mitophagy associated with an increase in cell apoptosis under nomaxia^76^. However, impaired synthesis of HIF-1α by inhibiting mTORC1/p70S6K/HIF-1α with melatonin leads to the disorder of autophagosome formation and the dysregulation of mitochondrial lysosomal co-location, which prevents cytoprotective mitophagy under hypoxia and enhances sorafenib lethality for Hep3B cells^77^.

### Autophagy and other multikinase inhibitors' resistance

The development of autophagy and resistance to multikinase inhibitors is not limited to sorafenib, and studies have confirmed the complex relationship between autophagy and the antitumor effect of lenvatinib and regorafenib^78,79^. Strikingly, nucleotide-binding oligomerization domain 2 activates the AMP-associated AMPK signaling pathway, resulting in an antitumor effect. This process activates the autophagy pathway and significantly increases the sensitivity of HCC cells to lenvatinib and sorafenib by directly binding with AMPKα–LKB1 complex, inducing apoptosis in the HCC mice model and xenograft tumor model^80^. Studies have revealed that the overexpressed TNFαIP8 blocks the AKT/mTOR signaling pathway and induces autophagy by direct interaction with ATG3–ATG7 proteins, rendering HCC cells more resistant to sorafenib and regorafenib^81^. Other studies have also demonstrated that the effect of lenvatinib is related to the ROS-dependent activation of ATM and inactivation of transcription factor elF2α, and found that higher levels of toxic autophagosome formation and lower levels of protective mitochondrial proteins are related to the cytotoxic effect of lenvatinib^82^.

### Autophagy and doxorubicin

Doxorubicin, as a first-line spectral antitumor drug, plays a cytotoxic role by inhibiting DNA replication and destroying the cell membrane structure and function. The nonselective cytotoxic molecule doxorubicin as the first-line drug for the treatment of HCC also falls into the dilemma of drug resistance, and the doxorubicin-induced autophagy is related to the drug-resistance mechanism^83^.

AKT inhibitor MK-2206 induces apoptosis and autophagy, but unfortunately, autophagy mediates drug resistance as a guardian of tumor cell survival. However, MK-2206 combined with doxorubicin has stronger cytotoxicity and tumor inhibition than doxorubicin alone in Mahlavu cells. There is no doubt that combination therapy has direct practical significance in inhibiting protective autophagy and promoting death^84^. The natural cycloartane triterpenoid (ADCX) derived from traditional Chinese medicine activates the AKT signaling pathway, inhibits the expression of lysosomal cathepsin B in doxorubicin-resistance HepG2/ADM cells, reduces the autophagy pathway, and further induces apoptosis^85^.

Likewise, the increase in autophagic vesicle and autophagic fluxes caused by doxorubicin was beneficial to cell survival under the treatment of doxorubicin in hepatoma Hep3B cells. This was further verified by the improvement of the cytotoxic effect of doxorubicin after inhibition of autophagy. The addition of (-)-epigallocatechin-3-O-gallate (EGCG) on the basis of doxorubicin could generate a stronger synergistic effect and lead to a stronger cytotoxic effect, which was closely related to LC3 expression inhibition and autophagy suppression^86^. However, the unicity of this experimental cell line (only HepG3) limits the promotion of EGCG and doxorubicin combination therapy, and the acquisition of detailed clinical data in other cell lines is necessary.

Another study developed ginsenoside Rg3 combination therapy with doxorubicin and made remarkable progress in reversing doxorubicin resistance in vitro and in vivo. Specifically, Rg3 sensitizes doxorubicin-induced cell death relating to the suppression of autophagic flux by the failure of degradation in the final stage of autophagy^87^. The effect of Korean Red Ginseng extract (RGE) on the amount of LC3-II and LC3 spots was inferred to inhibit the late autophagy flux. RGE combined with doxorubicin in the treatment of HCC serves as an effective strategy to make HCC become more sensitive to doxorubicin by inhibiting the autophagy flow in the final stage^88^.

### Autophagy and platinum drugs

Patients with advanced HCC benefit from treatment with platinum drugs, including cisplatin and oxaliplatin, while chemoresistance leads to a poor prognosis eventually. Cisplatin and oxaliplatin appear to induce autophagy of HCC, while tumor cells are significantly more sensitive to drugs after autophagy inhibition, which is significant for both cisplatin and oxaliplatin^62,89^.

From the perspective of the tumor microenvironment, tumor-associated macrophages (TAMs) inducing autophagy are conducive to the survival of HCC under oxaliplatin therapy. Inhibition of autophagy by ATG5 silencing technique can effectively improve the sensitivity of the coculture system of TAMs and HCC to oxaliplatin^90^.

A breakthrough has been made in the field of mitophagy and cisplatin resistance. Cisplatin therapy for HCC can activate autophagy and lysosomal biogenesis, which leads to mitochondrial–lysosomal crosstalk, and promotes cisplatin resistance by virtue of lysosome protection. However, PI3K/mTOR inhibitor PKI-402, resulting in lysosomal membrane permeabilization, may interfere with mitochondrial and lysosomal interactions and improve the chemotherapy killing effect of cisplatin^91^. Cisplatin maintained cell survival by activating mitophagy via dynamin-related protein 1 (DRP1), while DRP1 inhibitors upregulated Bax and downregulated Bcl-xl, leading to increased mitochondrial membrane permeability, blocking mitophagy, and promoting the release of cytochrome C, which was conducive to cisplatin-induced apoptosis^92^. The prospect of targeting mitophagy to promote apoptosis and thus enhance the efficacy of cisplatin for HCC has been recognized.

Autophagy activation measured by increasing the level of LC3 and autophagosome formation is significantly correlated with oxaliplatin resistance, while autophagy inhibition by ATG7 interference and chloroquine increased the sensitivity to oxaliplatin in HCC^93,94^.

### Autophagy and immune checkpoint blockade immunotherapy

With the rise of immune checkpoint blockade immunotherapy, patients with advanced HCC benefit greatly from immune checkpoint blockade (ICB) immunotherapy^95^. ICB immunotherapy inhibits tumor cell immune escape, destroys immune tolerance, and exerts anticancer effects via checkpoint-mediated inhibition of PD-L, PD-L1, and CTLA-4 (ref. ^96^). Furthermore, progress has been made in the application of ICB in HCC, such as anti-CTLA-4, anti-PD-1/PD-L1, a combination of both, and immune checkpoint inhibitors combined with surgical resection, multikinase inhibitors, chemotherapy, or local area therapies^95–98^. Despite the response rates of ICB rarely exceeding 20–25%, ICB appears to be one of the technical and conceptual breakthroughs in HCC treatment^99^.

In order to enhance the response to ICB therapy, much effort should be identified to overcome ICB resistance. However, the current understanding of ICB-based immunotherapy resistance is not very clear, especially the role of autophagy. Autophagy dysfunction damages the development of the immune system, inhibits T-cell proliferation and differentiation, promotes CD8^+^ T-cell aging, and suppresses the antitumor immunity in HCC^100^. Otherwise, the degradation of the major histocompatibility complex class I (MHC-I) by enhanced autophagy results in the impaired antigen presentation and ICB resistance in pancreatic ductal adenocarcinoma. This may provide a rationale for the combination of autophagy inhibition and ICB therapy in PDAC^101^ and makes a good example to overcome ICB resistance in HCC. Coincidentally, anti-PD-1 antibody combined with mTOR inhibitor restrains HCC growth than either single agent alone via affecting the combination of PD-1, eIF4E, and S6 (ref. ^102^). A study containing three independent cohorts of 578 HCC patients confirmed that higher PD-L1 expression was significantly and independently associated with an unsatisfactory survival in HCC patients^103^, and autophagy-related genes (ATG) have been identified as predictive signatures for anti-PD-L1 immunotherapy^104^. Furthermore, a combination administration based on a Listeria-based HCC vaccine, Lmdd-MPFG, and the anti-PD-1 immune checkpoint blockade antibody reveals the magic of autophagy. Mechanistically, Lmdd-MPFG activates the NF-κB signaling pathway of tumor-associated macrophages, affects the level of p62, and activates autophagy, enabling the tumor-center T cells to restore their sensitivity to anti-PD-L1 immunotherapy^105^. Although the existing research is quite limited, the regulation of autophagy has a very broad application prospect for improving the sensitivity of immunotherapy.

## Autophagy and CSCs in HCC drug resistance

By virtue of the unique biological characteristics and the interaction network with the tumor microenvironment, CSCs are a predominant contributor to drug resistance. The high expression of stemness markers like CD133, CD44, and the activation of stemness-related signaling pathways like STAT3, AKT, and NF-κB, promote the drug resistance of CSCs in HCC^106,107^. EMT, a dedifferentiation program converting non-CSCs to CSCs by imparting heritable phenotypic changes via epigenetic modifications, helps CSCs acquire drug-resistance ability under antitumor responses^108–110^. CSCs predominated in the G0 phase with a relatively inactive DNA replication and formed a cellular cluster in cancer foci, which helps to resist DNA damage and apoptosis caused by chemotherapy^111^. CSCs have a higher expression of ABC transporter, which facilitates drug excretion, reduces drug concentrations, and attenuates the damaging effects^112,113^. The interaction between the tumor microenvironment (TME) and CSCs also contributes to the CSC-mediated chemoresistance. TME exposing constantly to nutritional deprivation and hypoxia promotes CSC traits with increasing insensitivity to antitumor therapy^114–116^. Notably, tumor-associated macrophages secrete IL-6, activate STAT3 signal, and promote CSC proliferation, which is beneficial to chemotherapy resistance^117^.

Although the cognition of CSCs and drug-resistance mechanism has been expanded in HCC, there are still cognitive limitations on the role of autophagy in CSC-related drug resistance. Studies have revealed that CSCs developed protective autophagy in harsh microenvironments such as nutrient deprivation or hypoxia to maintain stemness, showed higher survival, less apoptosis, and higher clonogenic ability in CD133^+^ populations^8,118^. Lai et al. proved that TARBP2 protein is hydrolyzed by autophagic lysosomes, and the decrease in TARBP2 leads to the increase in the CSC marker Nanog, which promotes the development of sorafenib resistance in HCC cells^119^. Interferon-gamma (IFN-γ), as an important cytokine of the immune system to kill cancer cells, has been proved to induce tumor cell apoptosis or autophagy directly^120,121^. Furthermore, IFN-γ can induce autophagy in low CD133^+^ percentage cell lines, but not that in high CD133^+^ percentage cell lines. A difference in treatment results of IFN-γ was investigated in different HCC cell lines: a high percentage of CD133^+^ cells (Huh7 and PLC8024) were more resistant to IFN-γ treatment than HCC cell lines with a low percentage of CD133^+^ cells^122^. However, it is a pity that although the phenomenon “IFN-γ induced the autophagy of low CD133^+^ cell lines to decrease proliferation” has been found in this paper, the specific mechanism has not been fully proved. To further explore the mechanism of autophagy and drug resistance of CSC cells, more efforts need to be made at present. In addition, the development of efficient and accurate autophagy flux detection techniques and the separation of CSCs based on blood or tissue of clinical patients is also a huge bottleneck for predicting autophagy activation status and drug efficacy of patients.

## Autophagy and HCC-derived exosome in HCC drug resistance

Exosomes, as vesicles that mediate cellular communication, contain many components such as transcription factors, enzymes, nucleic acids, lipids, and extracellular matrix proteins. Tumor-derived exosomes mediate tumor microenvironment reconstruction, angiogenesis, invasion, and drug resistance in the progression of HCC, but the correlation between exosomes and autophagy has not been well explained in the mechanism of drug resistance^9,123^. Studies have involved a variety of biological processes, signaling pathways, and molecular mechanisms in miRNA regulation of tumor resistance, among which miRNA affects the expression of autophagy-related genes, and a breakthrough has been made in affecting drug resistance through the autophagy pathway^124^. It is gratifying to note that the mechanism by which exosome-derived miRNA regulates autophagy and influences drug resistance is gradually recognized. Exosomal miR-12 induced by acidic microenvironment promotes the malignant progression and miR-12 inhibits autophagy activity via the PTEN/AKT pathway, which is of great significance to the acquisition of sorafenib resistance^125,126^. Although the study did not further demonstrate the differences between exosome-derived miR-12 and total miR-12 in the regulation of cell resistance and malignant progression, there is no doubt that the regulation of autophagy by tumor cell-derived exosomes has profound research significance and bright application prospects.

## Autophagy and non-coding RNA in HCC drug resistance

Non-coding RNA (ncRNAs), a class of transcripts, including microRNA and long lncRNA without protein-coding capacity, are regarded as drug targets in many cancers^127^. A growing field of ncRNAs and autophagy provides new insights for the reversal of autophagy-related drug resistance in HCC (Table 1).

**Table 1:** Non-coding RNA in autophagy-related drug resistance of HCC.

Non-coding RNA	Drug administration	Alterations autophagy proteins in HCC	References
miR-26a/b	Doxorubicin	ULK1	^128^
miR-223	Doxorubicin	LC3, p62	^129^
miR-101	Cisplatin	ATG4D, STMN1, RAB5A	^130,131^
miR-199a-5p	Cisplatin	ATG7	^132^
miR-125b	Oxaliplatin	EVA1A	^133^
lncRNA HULC	Oxaliplatin, pirarubicin, 5-fluorouracil	USP22	^134^

As an important participant in autophagy initiation, ULK1 has been reported to be associated with doxorubicin sensitivity. Furthermore, 30 clinical specimens were tested and the level of miR-26a/b in tumor tissues was lower than that in para-carcinoma tissues, and only negatively correlated with the level of ULK1 protein, but not with the change of ULK1 mRNA level. The regulatory effect of miR-26a/b on the post-transcriptional level of ULK1 inhibits autophagy, promotes apoptosis, and makes HCC sensitive to doxorubicin chemotherapy, which is supported by detailed data in vivo and in vitro^128^.

A study in 2019 indicated that there was a lower miR-223 expression in HCC cells compared with normal liver cells, while overexpression of miR-223 directly caused the low expression of FOXO3a, which inhibited doxorubicin-induced autophagy levels by decreasing the LC3-II/LC3-I ratio while increasing p62 expression and caused doxorubicin resistance^129^. Coincidentally, overexpression of miR-101 negatively regulated the target gene EZH2, which can inhibit doxorubicin-induced autophagy, improve the cell-killing effect of doxorubicin, and induce cell apoptosis^130^. Similarly, interpretation of autophagy regulation at the level of ncRNA revealed that miR-101 inhibited autophagy via targeting ATG4D, STMN1, and RAB5A, and enhanced cisplatin-induced apoptosis of HepG2 cells^131^. In addition, the level of miR-199a-5p was significantly reduced in HCC patients treated with cisplatin, which promoted autophagy activation by targeting ATG7 and was conducive to cell survival under cisplatin treatment. In contrast, overexpression of miR199a-5p can inhibit autophagy and improve the efficacy of cisplatin^132^. Besides, miR-125b expression was downregulated in oxaliplatin-resistant HCC, while EVA1A expression, the target of miR-125b, was upregulated. The negative feedback relationship between miR-125b and EVA1A mediates autophagy-related oxaliplatin resistance, and overexpression of miR-125b effectively reverses oxaliplatin resistance via EVA1A-related autophagy^133^.

LncRNA HULC was significantly improved under oxaliplatin, 5-fluorouracil, or pirarubicin treatment and induced autophagy of liver cancer cells by stabilizing silent information regulator 1 (Sirt1) protein. The specific mechanism involves lncRNA HULC upregulating USP22, removing polyubiquitinated protein chains, and promoting Sirt1 ubiquitination degradation^134^. These data fully proved that the regulation of autophagy by ncRNA was a new way to regulate chemotherapy resistance^135^.

## Drug treatments plus anti-autophagy therapy in preclinical studies

Some achievements have been made in the basic research on autophagy and HCC drug resistance, and the clinical research is also in full swing (Table 2). One clinical trial that enrolled 68 patients with advanced HCC is studying if sorafenib/hydroxychloroquine (HCQ) will have improved efficacy when compared to sorafenib alone. In addition, the study aims to explore the addition of HCQ to reverse sorafenib tolerance in patients and lead to HCC stability. A phase I study of MLN9708 (proteasome inhibitor) and vorinostat (HDAC inhibitor) to target autophagy in patients with advanced p53 mutant malignancies is to find the highest tolerable dose of the combination of MLN9708 and vorinostat that can be given to patients via a single-group assignment. Another clinical trial aims to find the highest tolerable dose of sirolimus (mTOR inhibitor) or vorinostat that can be given in combination with autophagy inhibitor HCQ in 143 patients with advanced cancer. This phase I trial also studies the safety of sirolimus or vorinostat in combination with HCQ and lays the foundation for the follow-up research. Furthermore, an observational study would like to study whether autophagy biomarker sirtuin1 plays a cytoprotective role in liver injury. A liver resection/liver transplantation surgery has been scheduled in 41 participants with liver cancer or hepatobiliary tract adenomas and carcinomas and a small piece of tissue would be removed to undergo additional laboratory testing. Even though the mechanism of the sirtunin1-autophagy pathway in drug resistance has not been investigated, the role of the sirtunin1-autophagy pathway in orthotopic liver transplantation^136^ and oxidative stress^137^ has been studied preliminarily. These data arouse researchers’ curiosity about the mechanism of the sirtunin1-autophagy pathway in drug resistance of HCC. More efforts should be made to develop therapeutic strategies based on autophagy inhibition to improve the hepatic function of patients with liver diseases.

**Table 2:** Clinical trials involving autophagy in advanced cancers, especially in HCC.

Trials identifier	Phase	Status	Design	Patient population	Study type	Model	Enrollment
NCT03037437	Phase 2	Recruiting	Sorafenib hydroxychloroquine	Hepatocellular cancer	Interventional (clinical trial)	Parallel assignment	68 participants
NCT02102971	/	Terminated	Surgical operation	Liver cancer hepatobiliary tract adenomas and carcinomas	Observational	Cohort	41 participants
NCT01266057	Phase 1	Active, not recruiting	Hydroxychloroquine sirolimus vorinostat	Advanced cancers	Interventional (clinical trial)	Single group assignment	143 participants
NCT02042989	Phase 1	Active, not recruiting	MLN9708 vorinostat	Advanced cancers	Interventional (clinical trial)	Single group assignment	68 participants

The information of Table 2 was obtained from http://clinicaltrials.gov/.

## Conclusion

**Fig. 3:**
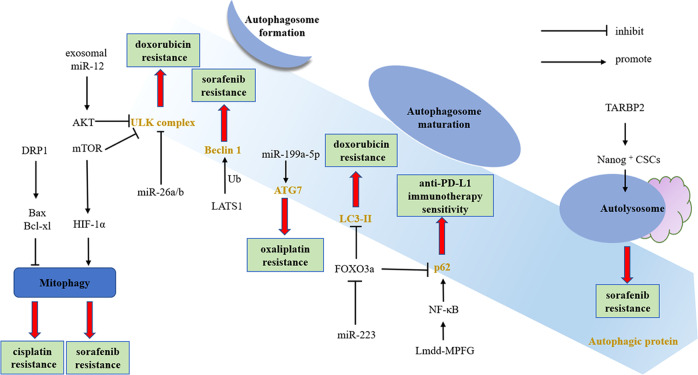
Drug resistance and autophagy in HCC. The exosomal miR-12 regulates AKT to inhibit doxorubicin resistance. MiR-26a/b negatively regulates the ULK complex, inhibits autophagy, and reverses doxorubicin resistance. The expression of Beclin 1 is upregulated by sorafenib therapy, but Beclin 1 ubiquitinated by LATS1 inhibits autophagy, thereby promoting sorafenib resistance. HIF-1α regulates mitophagy to promote sorafenib and cisplatin resistance. The high expression of miR-223 downregulated the expression of FOXO3a, inhibits autophagy levels through LC3 and p62, and promotes doxorubicin resistance. Activation of NF-κB induces autophagy by regulating the level of p62 and enhances the sensitivity of anti-PD-L1 immunotherapy. DRP1 inhibits mitophagy through Bax and Bcl-xl and reverses the occurrence of cisplatin resistance. TARBP promotes the expression of stemness marker Nanog in CSCs, facilitates the fusion of lysosomes and autophagosomes, and promotes sorafenib resistance.

HCC, which refers to the major subtype of liver cancer, has caused thousands of people to roll in death. Unfortunately, many patients especially those with advanced HCC develop drug resistance and result in an intractable conclusion. Recent data suggest that autophagy tends to play an accomplice role in drug resistance and the interface between the two is multifactorial and crosstalk occurs at different proteins of each process. The expression dysregulation of Beclin 1, p62, or LC3- induced autophagy activation is explored to contribute to drug resistance. Furthermore, the regulation of autophagy by targeting autophagy-related proteins, signaling pathways, CSCs, HCC- derived exosome, and ncRNA is conducive to the reversal of drug resistance (Fig. 3). All in all, the combination of autophagy inhibitors and molecular targeted or chemotherapy drugs has miraculously achieved better therapeutic effects in vivo and in vitro. However, there are still many difficulties in the exploration of the combination of autophagy inhibition with existing HCC therapies: the variability of the course of the disease, the complexity of the autophagy mechanism, and the individualized requirements of treatment. A deeper understanding of the autophagy in drug resistance is conducive to improving the sensitivity of HCC to antitumor drugs and more multicenter clinical trials are required for anti-HCC therapy combined with autophagy inhibition.
